# Anatomical MRI and [^18^F]FDG PET/CT imaging of *Schistosoma mansoni* in a NMRI mouse model

**DOI:** 10.1038/s41598-020-74226-2

**Published:** 2020-10-15

**Authors:** Tobias Lindner, Jan Stenzel, Nicole Koslowski, Alexander Hohn, Änne Glass, Sarah M. Schwarzenböck, Bernd J. Krause, Brigitte Vollmar, Emil C. Reisinger, Martina Sombetzki

**Affiliations:** 1 Core Facility Multimodal Small Animal Imaging, Rostock University Medical Center, Rostock, Germany; 2 Division of Tropical Medicine and Infectious Diseases, Center of Internal Medicine II, University Medical Center Rostock, Rostock, Germany; 3 Department of Nuclear Medicine, Rostock University Medical Center, University of Rostock, Rostock, Germany; 4 Institute for Biostatistics and Informatics in Medicine and Ageing Research, Rostock University Medical Center, University of Rostock, Rostock, Germany; 5 Institute of Experimental Surgery, Rostock University Medical Center, University of Rostock, Rostock, Germany

**Keywords:** Parasitic infection, Experimental models of disease

## Abstract

Schistosomiasis represents one of the most devastating worm parasitosis in the world. Current diagnostic methods are insufficient to determine the infection grade and the disease related organ damage. We herein investigated whether discrimination of infection grade and its correlation to liver damage could be accurately performed by multimodal imaging in a mouse model of *Schistosoma mansoni* infection. Therefore, groups of uninfected and infected mice underwent MRI and [^18^F]FDG PET/CT imaging. Anatomical MRI images were used for liver volumetry and for quantification of hepatic granulomas. For PET/CT images a volume of interest based analyses were employed to calculate the [^18^F]FDG uptake in liver, portal vein, spleen and abdomen. Herein, we demonstrate that the combined use of [^18^F]FDG-PET/CT and MRI represents an appropriate diagnostic tool for *Schistosoma mansoni* infection, but fails to discriminate the infection grade and the linked organ damage. Only the splenic [^18^F]FDG uptake in the 25 cercariae group (5.68 ± 0.90%ID/cc) and 50 cercariae group (4.98 ± 1.43%ID/cc) was significantly higher compared to the control group (2.13 ± 0.69%ID/cc). Nevertheless, future multimodal imaging studies with new radiopharmaceuticals could build a highly sensitive and specific basis for the diagnosis and evaluation of organ damage of schistosomiasis.

## Introduction

Schistosomiasis is caused by digenetic blood trematodes of the genus *Schistosoma spp.* and represents one of the most devastating worm parasitosis in the world, accounting for more than 260 million infected people, mainly in tropic and sub-tropic regions^1^. In developing countries the disease has a significant impact on economic and public health^2^. The main human pathogenic species of schistosomes are *Schistosoma mansoni* (*S. mansoni*)*, Schistosoma japonicum* (*S. japonicum*) and *Schistosoma haematobium* (*S. haematobium*)^3^. People become infected by skin contact to waterborne cercariae shed by infected fresh water snails^3,4^. In the body the cercariae undergo a variety of morphological changes and transform into juvenile larvae known as schistosomula^5^. Schistosomula invade blood vessels and migrate through the heart and the lung into the vascular system of the liver, where they mature into adult male and female worms. After maturation, paired worms migrate against the blood flow to the veins of the small intestine (*S. japonicum*), colon (*S. mansoni*) or bladder (*S. haematobium*)^6^. Here, the female parasites produce several hundred eggs per day^7^. Parasite eggs provoke local inflammatory lesions within the vessel wall which pave the way to reach the gut or the bladder wall to be finally shed into the environment through feces or urine of the host^8^. Parasite eggs contain miracidia that hatch by fresh water contact to infect fresh water snails for completion of developmental cycle of schistosomes^2,8^. However, most of the parasitic eggs (*S. mansoni* and *S. japonicum*) are flushed back to the liver via bloodstream to become entrapped within the small liver sinusoids. Here they provoke T-cell mediated inflammation resulting in the generation of granulomatous lesions followed by vigorous fibrous tissue formation^1,9^. After long-term infection (5–20 years) chronic progressive liver fibrosis leads to portal hypertension and its clinical sequelae, such as ascites and gastrointestinal/esophageal varices bleeding associated with high mortality rate^8–13^.

Currently, the gold standard therapy is based on the anthelmintic drug Praziquantel. It eliminates adult worms, but does not cure chronic liver or intestinal damage and provides no protection against future or recurrent infections^14^. Moreover, mass drugging with Praziquantel paves the way for emergence of Praziquantel resistant schistosome strains^15^. Steinemann et al. have estimated more than 779 million people worldwide holding a risk for infection with schistosomes in future^16^.

Besides the urgent need for new therapeutic options, it is necessary to have thorough assessment of the disease progression and evaluation of drug effectiveness of future therapy applications against schistosomiasis. In clinical practice, parasitological techniques such as filtration of urine and examination of stool (microscopy-based Kato-Katz or Telemann) are still the methods of choice for diagnosis and monitoring of drug efficacy. But these methods have low sensitivity, especially in low-prevalence areas, have a significant time-delay as they are dependent on eggs and therefore do not detect early infections and cannot accurately display the disease burden^6,17–19^. Immunodiagnostic techniques are in fact more sensitive and easier to use, but antibodies can be detected even years after infection. This makes it difficult to distinguish between active and previous exposure^17,20–22^. DNA (circulating cell-free parasite DNA) and RNA (circulating miRNAs) based diagnostic methods have been proven to be highly sensitive and specific^23^. The requirement for specialized laboratories makes the broad field application of these methods a problematic issue. In addition, the detection of the intensity of infection as an indicator of morbidity^24^ and parameter for drug effectiveness is barely covered by any of the above methods.

Modern imaging techniques like positron emission tomography / computed tomography (PET/CT) and magnetic resonance imaging (MRI) could improve diagnostic of schistosomiasis, in particular with a focus on assessing the degree of infection, and could help to evaluate the efficiency of novel immunoprotective applications during the course of the disease. Due to the high metabolism of glucose by the parasite worms^25^, Salem et. al demonstrated that the PET imaging with [^18^F]FDG as radiotracer is a useful tool to directly detect large numbers of the parasites themselves in infected animals^20^. In addition, they also showed a strong correlation between [^18^F]FDG uptake in the portal vein area and worm burden when more than 50 worms are present^20^. Moreover, the characterization of a mouse model of schistosomiasis using MRI demonstrated that this in vivo imaging technique is capable of monitoring liver damage during the course of the disease^26^. The pathological findings obtained in this MRT study were consistent with known characteristics of infected people, confirming the convenience of MRI imaging as an appropriate instrument to detect schistosomiasis and determine the efficacy of new developed therapies^26^. In light of these results, we assume that a combined use of PET/CT and MRI could be a beneficial technique for clinicians and researchers to visualize and characterize the worm load in infected individuals and to evaluate new treatments for schistosomiasis in vivo.

Therefore we herein performed for the first time a multimodal [^18^F]FDG-PET/CT and anatomical MRI study in a mouse model of *S. mansoni* to investigate the usability of this combination for determining the degree of infection and its correlation to possible tissue or organ damage. In addition, the results of multimodal imaging were validated in relation to the histological findings.

## Results

### MRI

**Figure 1:**
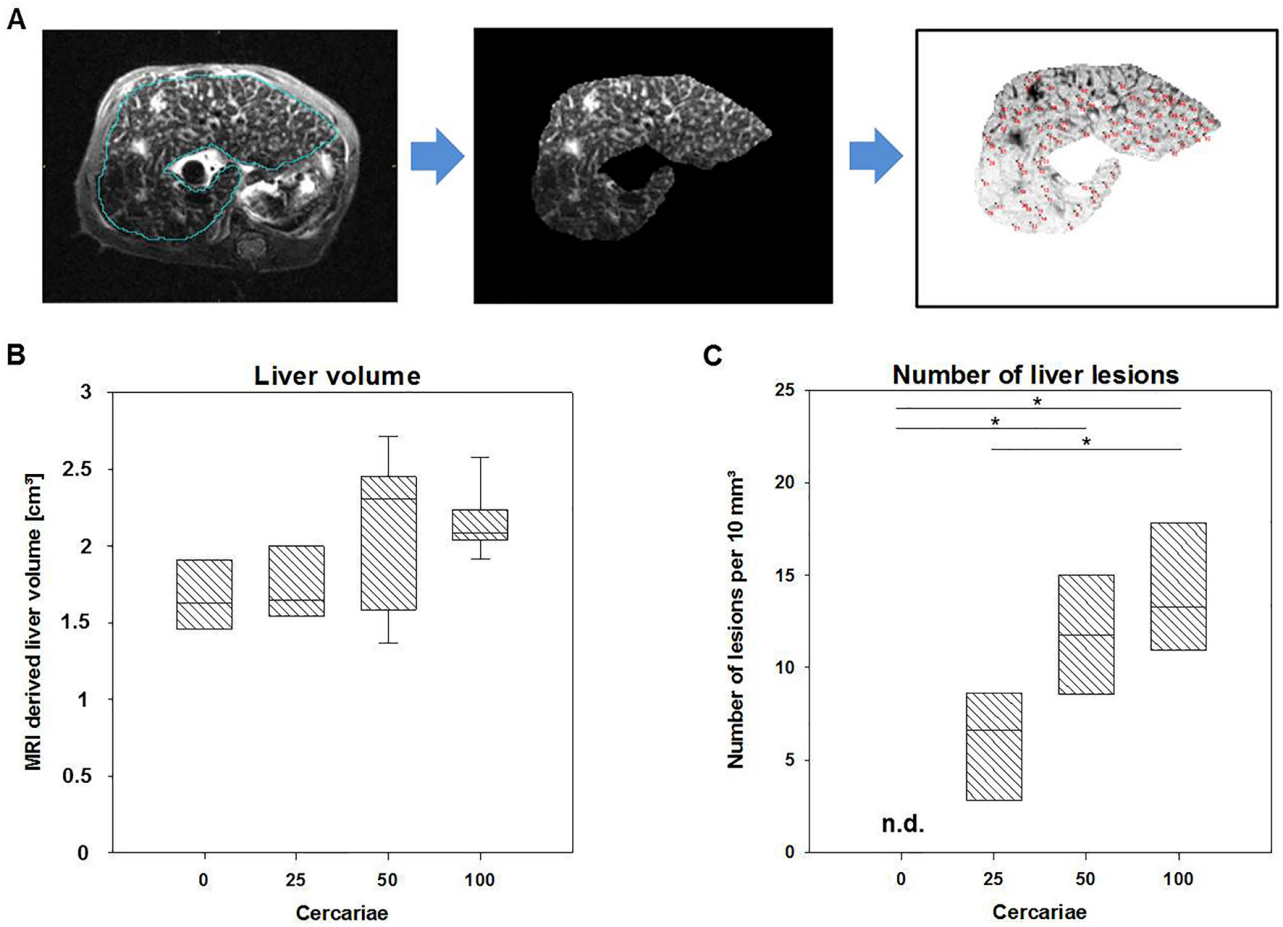
Quantification of liver volume and number of hepatic lesions, in uninfected and infected mice with different infection intensities of *Schistosoma mansoni*. (**A**) T2 weighted axial MRI image (TurboRARE-Sequence) with segmentation of the liver (top left); cropped liver slice (top middle); inverted contrast image of the cropped liver slice for better detectability of the lesions (top right). Manually counted lesions in the liver slices (red dots). (**B**) Liver volumes in the different groups derived from T2-weighted MRI show no significant difference (*p* = 0.282). (**C**) Number of liver lesions per 10 mm^3^ counted on three consecutive T2-weighted MRI slices of the liver. (n.d.—not detectable).

**Figure 2:**
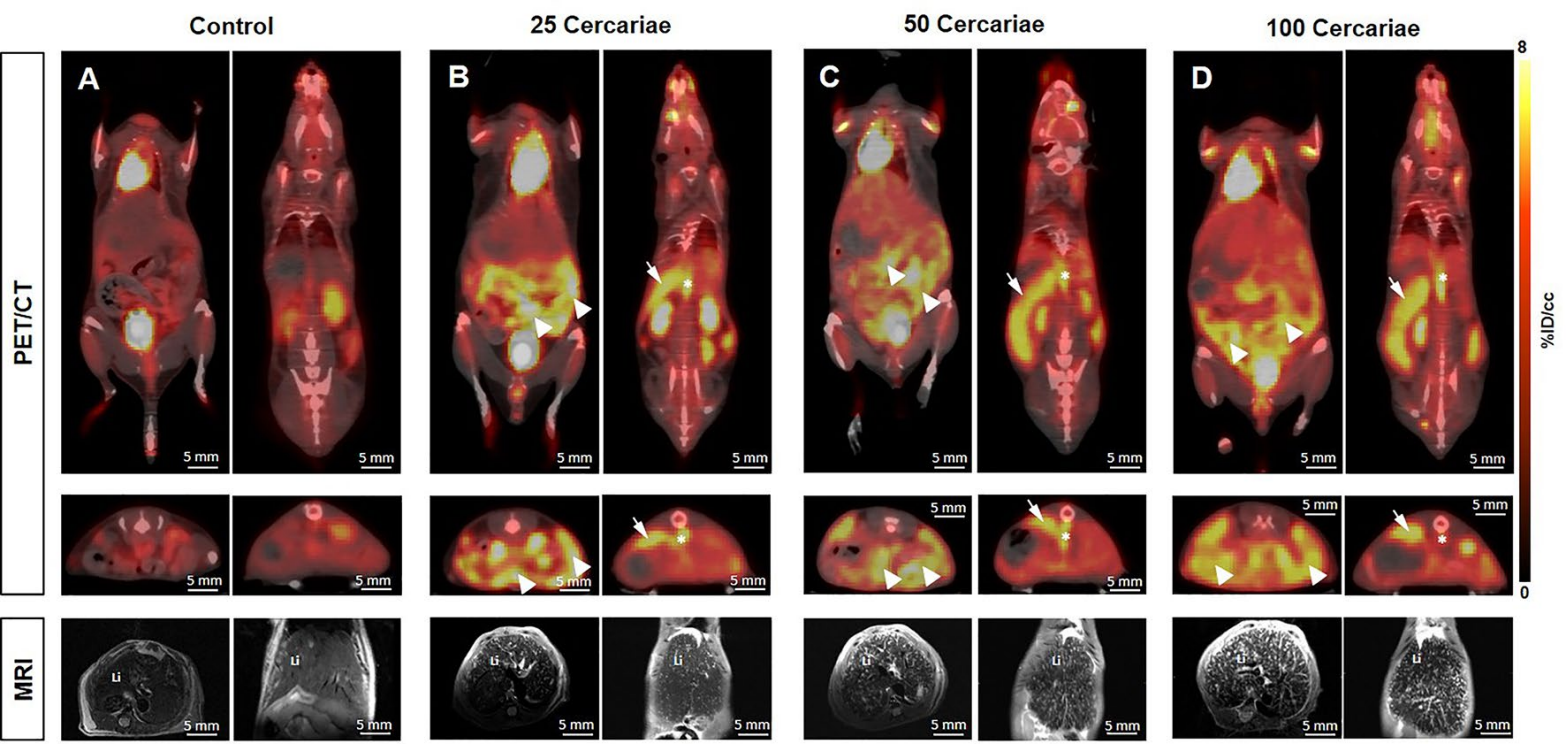
[^18^F]FDG-PET/CT- and MRI imaging of uninfected and infected mice with different number of *Schistosoma mansoni* cercariae. Representative coronal and axial [^18^F]FDG/CT- and T2-weighted MRI images of (**A**) uninfected and with (**B**) 25 cercariae (**C**) 50 cercariae or (**D**) 100 cercariae infected mice. Upper row illustrates the elevated [^18^F]FDG uptakes in abdomen (arrowheads), portal vein (asterisks) and spleen (arrows) of infected mice compared to the uninfected mice in ventral (left) and dorsal (right) coronal slices. The middle row shows the corresponding axial images. An infection grade dependent increase in the number of granulomatous lesions of the liver (Li) is depicted in axial (left) and coronal (right) MRI slices (bottom row).

**Figure 3:**
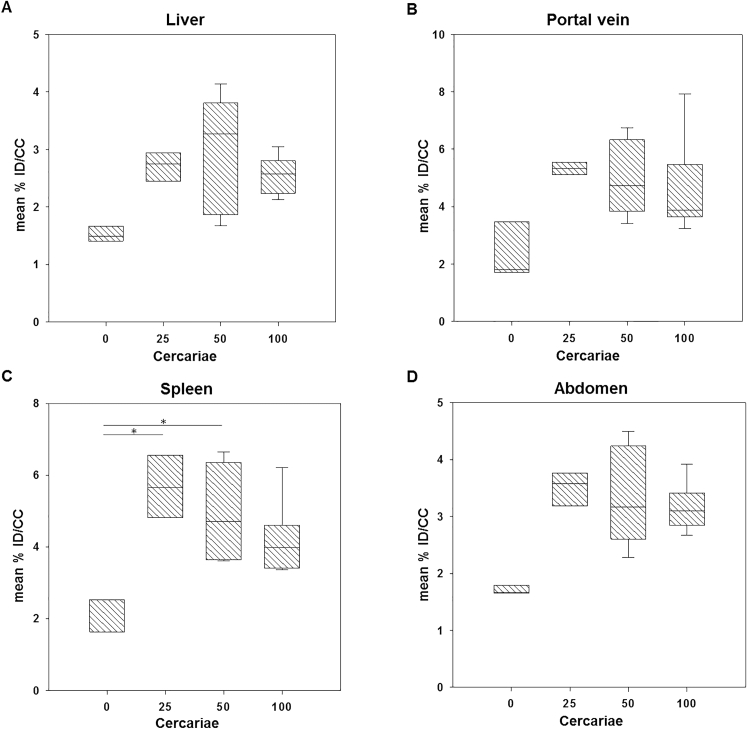
Quantification of [^18^F]FDG uptake in uninfected and infected mice with different number of *Schistosoma mansoni* cercariae. Mean %ID/cc in (**A**) liver, (**B**) portal vein, (**C**) spleen and (**D**) abdomen of uninfected and infected mice with different infection grade. All four examined regions revealed comparable [^18^F]FDG uptakes among infected groups which were higher compared to the uptake of the control group. However, significant differences have been observed only for the spleen (control vs 25 and 50 cercariae).

In all infected groups (25, 50 and 100 cercariae) anatomical MRI (Figs. 1 and 2) revealed signs of mouse schistosomiasis as described by Kosaka et al. and Masi et al.^26,27^ six weeks post infection. The MRI derived mean value of the liver volume (Fig. 3A) was comparable in all four groups. Animals with higher worm burden did not show significant signs for hepatomegaly (liver volume: 0 cercariae (control): (mean ± SD: 1.67 ± 0.31) cm^3^, 25 cercariae: (1.77 ± 0.33) cm^3^, 50 cercariae: (2.08 ± 0.55) cm^3^, 100 cercariae: (2.16 ± 0.25) cm^3^; *p* = 0.282).

Quantification of multifocal hyperintensities (lesions) in the liver (Fig. 1B) has shown a correlation between the number of lesions and the degree of infection. The highest amount of lesions was found in the group infected with 100 cercariae (14.15 ± 4.18 lesions per 10 mm^2^), compared to the group infected with 25 cercariae (6.01 ± 3.19 per 10 mm^2^, *p* = 0.016—25 cercariae vs. 100 cercariae) and 50 cercariae group (11.77 ± 3.34 per 10 mm^2^, *p* = 0.716—50 cercariae vs. 100 cercariae). The groups 50 and 100 displayed a significant difference in number of lesions compared to the uninfected mice. (*p* < 0.001 for control vs. 100 cercariae and *p* = 0.003 for control vs. 50 cercariae).

### PET/CT

**Figure 4:**
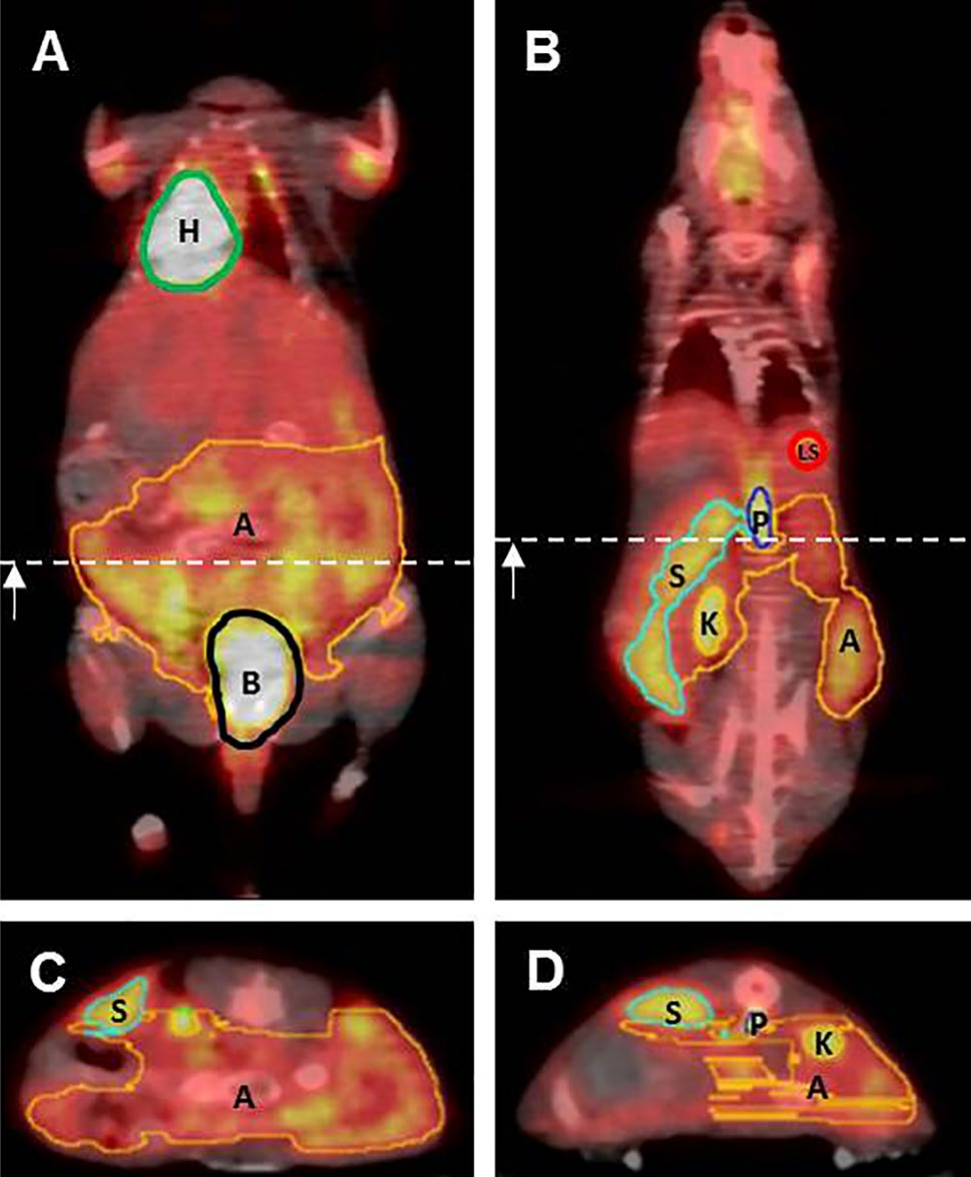
Illustration of VOI’s placement in [^18^F]FDG PET/CT images. Upper row shows placement of VOI’s in coronal faction of (**A**) a ventral slice and (**B**) a more dorsal slice. Dotted white lines display position of slice in (**C**) and (**D**). Bottom row shows slices of axial faction**.** VOIs: heart (H and green line), abdomen (A, orange line), bladder (B, black line), spleen (S, light blue line), portal vein (P, blue line), kidney (K, yellow line) and the liver sphere (LS, red circle).

With regard to the PET/CT images, high radioactive uptakes were observed in the heart, bladder and kidneys of control and infected mice (Fig. 4). However, compared to the controls, the infected mice showed further regions of intensive radioactive accumulation including the abdomen (arrowheads), portal vein (asterisks) and spleen (arrows) (Fig. 4B–D). Qualitative analysis of the PET/CT images showed slightly more radioactive intensity in the liver of infected mice compared to control mice.

For analysis of correlation between the infection burden and the [^18^F]FDG uptake, the % injected dose per cubic centimeter (%ID/cc) was calculated in the liver, portal vein, spleen and abdomen of each animal. In general, the total uptake of [^18^F]FDG in all investigated regions was elevated in infected mice compared to uninfected animals, whereas the radioactivity levels in the particular regions were quite similar between the groups of mice infected with different numbers of cercariae (Fig. 3).

Livers of control mice (mean 1.53 ± 0.18%ID/cc) displayed a lower uptake of [^18^F]FDG than infected mice, ranging between 66 and 92%: 25 cercariae: (mean ± SD) 2.69 ± 0.31%ID/cc; 50 cercariae: 2.94 ± 1.09%ID/cc, and 100 cercariae: 2.55 ± 0.36%ID/cc (Fig. 3A). We found similar results for the portal vein of the animals. Here, the uninfected mice showed a mean [^18^F]FDG uptake of 2.50 ± 1.31%ID/cc. The mice infected with 25, 50 as well as 100 cercariae revealed higher means with 5.33 ± 0.26%ID/cc; 5.01 ± 1.43%ID/cc and 4.70 ± 1.87%ID/cc respectively (Fig. 3B). Also, in the abdomen, an elevated [^18^F]FDG uptake was detected in the infected groups: 25 cercariae (3.48 ± 0.37%ID/cc), 50 cercariae (3.36 ± 0.94%ID/cc) and 100 cercariae (3.17 ± 0.47%ID/cc) were all nearly twofold higher than in control animals (1.72 ± 0.10%ID/cc) (Fig. 3D). All uptake differences found in liver, portal vein and abdomen between infected mice and controls could not detected to be statistically significant. Only the splenic [^18^F]FDG uptake in the 25 cercariae group (5.68 ± 0.90%ID/cc, *p* = 0.008) and 50 cercariae group (4.98 ± 1.43%ID/cc, *p* = 0.025) was significantly higher compared to the control group (2.13 ± 0.69%ID/cc), whereas mice infected with 100 cercariae mice did not exhibit significant difference from control (4.21 ± 1.16%ID/cc) (Fig. 3C). In each of the four regions investigated, the average [^18^F]FDG uptake of the stagewise infected mouse groups was comparable.

### Correlation between MRI and PET/CT

**Figure 5:**
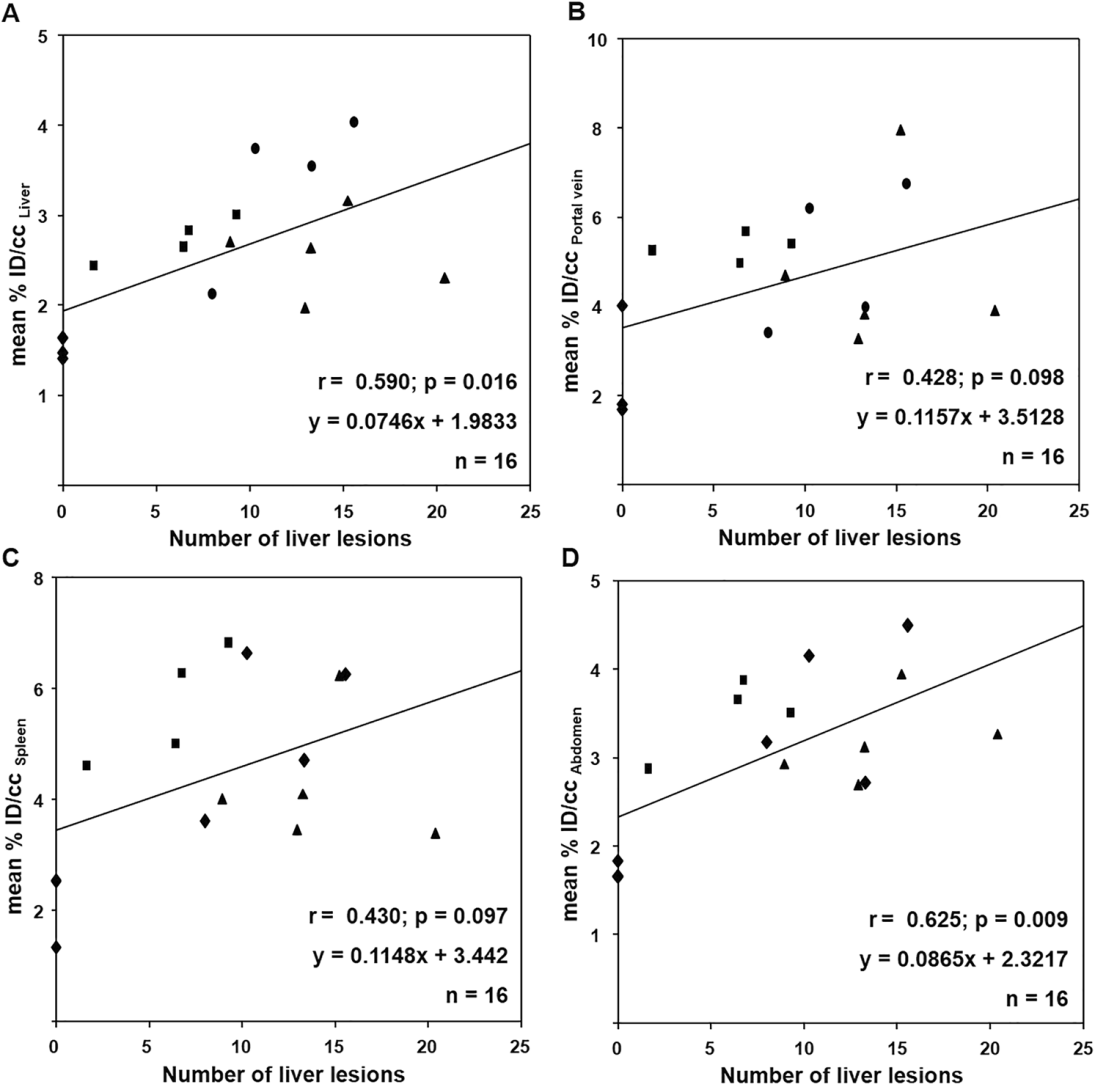
Quantitative analysis of correlation between [^18^F]FDG uptake and number of liver lesions. Relationship between the mean [^18^F]FDG uptake in (**A**) liver, (**B**) portal vein, (**C**) spleen, (**D**) abdomen and the number of liver lesion. The [^18^F]FDG uptake measured by PET/CT graphed as a function of the number of liver lesions measured by MRI. A linear correlation between the mean [^18^F]FDG uptakes of liver as well as abdomen and the number of liver lesions were found, whereas the radioactivity within spleen and portal vein shown no correlation to the number of liver lesions. All datasets were obtained from uninfected mice (diamond) and mice infected mice with 25 (square), 50 (circle) and 100 (triangle) cercariae.

To correlate PET/CT and MRI findings, the mean [^18^F]FDG uptake of livers, portal veins, spleens and abdomen was presented as a function of the number of MRI derived liver lesions (Fig. 5). Significant correlation between [^18^F]FDG uptake and the number of liver lesions was detected for both, liver (r = 0.590; *p* = 0.0162; n = 16; Fig. 5A) and abdomen (r = 0.625; *p* = 0.00962; n = 16; Fig. 5D). For portal vein (r = 0.428; *p* = 0.0978; n = 16) and spleen (r = 0.430; *p* = 0.0965; n = 16), the correlations could not be proven (Fig. 5 B and C). Positive slopes of linear regression lines show, for all regions likewise, increasing [^18^F]FDG uptake accompanied by increasing number of liver lesions; statistically significant both in liver and abdomen regions.

### Infection-related organ alterations and liver histology

**Figure 6:**
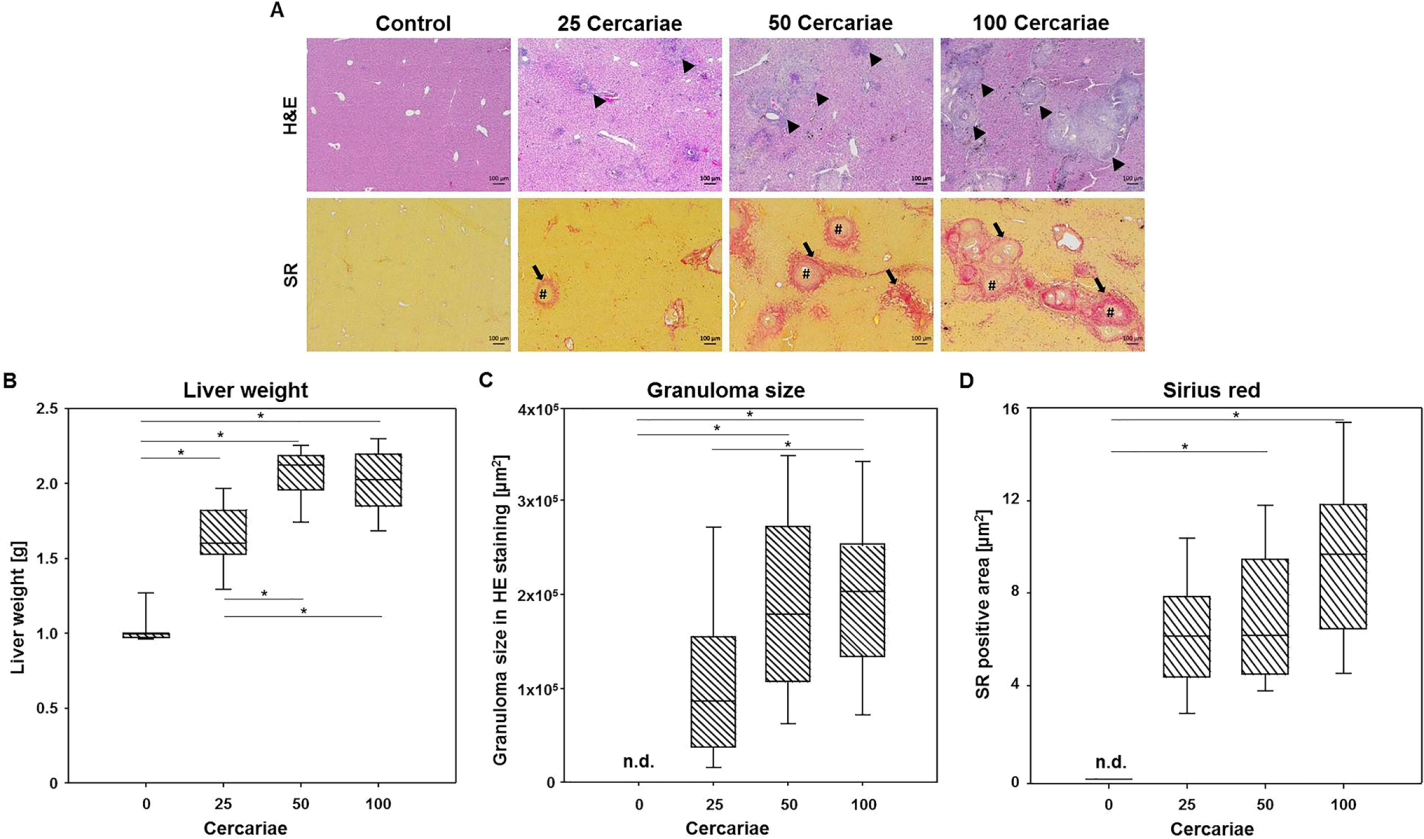
Infection-related organ alterations and liver histology. (**A**) Representative liver histology images of uninfected and infected mice with different number of *S. mansoni* cercariae (upper row—haematoxylin/eosin stain—(H&E); bottom row—Sirius red staining—(SR); original magnification (100x) are shown. H&E staining highlighted the hepatic granulomas (arrowheads) and Sirius red illustrates the extracellular matrix depositions of collagen (arrows) within the granulomas (#) in the injected mice. (**B**) Infection of mice with either 25 (n = 8), 50 (n = 8) and 100 (n = 8) *S. mansoni* cercariae resulted in a significant increase of liver weight compared to control mice (n = 6), whereas mice infected with 50 and 100 *S. mansoni* cercariae showed a significant higher liver weight than mice infected with 25 cercariae. (**C**) Morphometric analysis of granuloma size revealed a significant increase following infection with 50 and 100 *S. mansoni* cercariae compared to control mice and between the groups infected with 25 and 100 cercariae. (**D**) The area of hepatic fibrosis was significantly increased in livers of mice infected with 50 and 100 cercariae each compared with the control.

The total hepatic egg load was determined for each infection grade (25 cercariae: (4.6 ± 2.0) × 10^3^; 50 cercariae: (8.6 ± 2.6) × 10^3^; 100 cercariae: (20.2 ± 13.0) × 10^3^). The average egg load increased with the higher degree of infection. Significant, however, was only in the group of 100 cercariae comparede to 25 cercariae (*p* < 0.05). Furthermore, histological images revealed pathological alterations in the liver tissue of infected mice compared to controls (Fig. 6 A). All groups of infected animals displayed a significant increase in liver weight (25 cercariae: (1.64 ± 0.25) g, *p* < 0.001; 50 cercariae: (2.06 ± 0.19) g, *p* < 0.001; 100 cercariae: (2.01 ± 0.23) g, *p* < 0.001) compared to the healthy controls (1.04 ± 0.13) g. Moreover, mice infected with 50 (*p* = 0.002) and 100 (*p* = 0.007) *S. mansoni* cercariae displayed a significantly higher liver weight than mice infected with 25 *S. mansoni* cercariae (Fig. 6 B). While the livers of uninfected mice were free of granulomas, we found increasing granulomatous affected areas with the infection grade: 25 cercariae (1.14 ± 0.25) × 10^5^ µm^2^, 50 cercariae (1.91 ± 0.51) × 10^5^ µm^2^, and 100 cercariae (2.00 ± 0.34) × 10^5^ µm^2^. Significant differences were detectable between infected groups of 25 and 100 cercariae and between control and 50 cercariae as well as control and 100 cercariae (*p* < 0.05) (Fig. 6 C). The quantification of collagen deposition within the liver revealed a comparable result. Highest levels of Sirius red positive areas were found in mice infected with 100 cercariae (9.83 ± 3.57) µm^2^, compared to the other experimental groups (25 cercariae: (6.24 ± 2,83) µm^2^; 50 cercariae: (6.70 ± 2.66) µm^2^), although the values do not reach statistical significance (Fig. 6 D).

## Discussion

In addition to the diagnostics of schistosomiasis, the assessment and quantification of worm and egg burden as well as the evaluation of infection-related tissue damage is a challenge^28^. Direct detection methods like Kato-Katz or Telemann stool microscopy or indirect detection of anti-bodies against schistosome antigens in the blood serum^29^ are not suitable for drawing conclusions about parasite associated organ damage. In human infection there is no gold standard for the evaluation of portal fibrosis^30^. Wedge or percutaneous liver biopsies are invasive methods that are unsuitable for long-term or follow-up assessment. Ultrasound scans or elastography methods have proven to be more suitable for weighing periportal fibrosis in a human schistosome infection^31,32^. For decades, the murine model of *S. mansoni* infection is used for experimental research and development of anti-fibrotic drugs or vaccines against the parasite. None of the above-mentioned detection methods used in humans are suitable or available for determination of drug effectiveness in long-term follow-up studies. In this study, we represent a multimodal imaging examination including PET/CT and MRI in a *S. mansoni* mouse model to weigh the potential of this combination for quantification of the infection burden and to monitor schistosomiasis-induced pathology. To our knowledge, this is the first time that such a combined in vivo imaging approach of *S. mansoni* has been applied. Furthermore, anatomical MRI and PET/CT data were cross-correlated with histological analyses. Overall, with our multimodal imaging study at the described murine model of *S. mansoni* infection we found diseases related pathological differences between uninfected and infected mice. In line with a previous MRI study^27^ that investigated the liver in a *S. mansoni* mouse model, we could also identify multifocal hyperintensities in anatomical T2-weighted images which are associated with granulomas within the liver of *S. mansoni* infected mice. These detectable hepatic granulomas are caused by the deposited parasite eggs which are produced by mate worm pairs living mainly in the veins of the colon and then flushed back to the liver via the blood stream^6,33^.

Mice infected with 100 cercariae displayed the highest number of hepatic granulomas. However, a significant difference was quantifiable only between light (25 cercariae) and high infection (100 cercariae) by the presented imaging analysis. This finding suggests that the T2-weighted MRI imaging is not sensitive enough to differentiate all grades of infection via quantification of egg granulomas in the liver. The experimental design chosen here is fairly artificial. We aimed here to cause three different degrees of liver damage in terms of the number of granulomatous lesions within the livers. We paid special attention to the different, widely spread infection intensities in order to investigate whether these differences can be quantified by MRI or PET/CT. We have used the system of 25, 50 and 100 cercariae in the past to analyze mild, moderate and severe clinical picture^34^. However, histological analyses of the granuloma sizes and collagen fibers within the livers using HE and SR staining revealed comparable results. These findings underline the assumption that beside the worm burden other factors like host genetic background, conditions of infection and parasite strains must play a role in the development of disease severity during *S. mansoni* infection^13^. Moreover, we observed a high inter-individuality regarding the disease progression and its representation in our MRI data analysis.

While an increase in liver weight was detected ex vivo, our volumetric analyses of the liver derived from the anatomical T2-weighted MRI images showed no significant increases of liver sizes as signs of hepatomegaly, typical for schistosome infection. According to the data of the liver volume measurement the infection intensity was not distinguishable. These discoveries are contrary to the findings of a previous MRI study with *S. mansoni* infected mice. Masi and colleagues have shown an increase in liver volume of 19% six weeks after infection with 30 cercaria of a Venezuelan *S. mansoni* strain^26^. This discrepancy between increased liver weight and steady liver volume found in our study could be explained by the disease related liver tissue alterations (e.g. formation of fibrosis or deposition of eggs) which are accompanied by density changes^35^.

Our results of the MRI analysis during *S. mansoni* infection suggest that the determination of the hepatic granulomas could serve as a marker to distinguish between uninfected and infected patients in clinical practice. However, in agreement with our histological analyses a classification of the infection grade was not clearly possible based on the MRI derived data of the liver. From our point of view, future studies on a larger cohort of animals and additional examinations of *S. mansoni* infection affected organs for example spleen and intestines^26^ are needed to establish MRI as appropriate diagnostic tool for quantitative assessment of disease burden. Furthermore, mri techniques which probe functional and quantitative parameters beyond anatomical imaging and T2/T2* relaxometry like perfusion and diffusion imaging should be investigated regarding their potential to determination the infection grade during *S. mansoni* infection.

PET imaging revealed an increased [^18^F]FDG accumulation following infection. Livers, portal veins, spleens and abdomen represent the primarily affected regions by schistosome infection. An elevated [^18^F]FDG uptake was detectable in all infected mice compared to the healthy controls. This observation goes in line with a previous PET study which also showed a disease related higher uptake of [^18^F]FDG in the abdomen, livers and portal veins of *S. mansoni* infected mice^20^. However, additional to this work we could identify a higher splenic tracer accumulation. Although it has been demonstrated that adult worms can import [^18^F]FDG as analogue of glucose due to their high glucose metabolism^20,36^, we presume that the determined elevated uptake of [^18^F]FDG in the infected mice was primarily driven by the innate inflammatory response of the mice directed against the parasites. The significant increase of [^18^F]FDG found in spleens of infected mice confirms this assumption due to the fact that the spleen is an immune active organ^37^ and that adult *S. mansoni* worms as well as parasite eggs are absent in the splenic tissue^38^. Similar to the MRI findings the quantification of the tracer distribution exhibited no significant differences in the uptake of [^18^F]FDG in our mice with different infection intensities. In contrary to this result, Salem et al.^20^ reported in an PET imaging study of *S. mansoni* infected mice a positive linear correlation between the total [^18^F]FDG uptakes in the portal veins as well as livers and worm burden. However, the ability to quantify the worm burden based on this relationship was limited in mice with low worm burden and only powerful in mice with more than 50 worms^20^. Since only approx. 60% of the penetrated cercariae mature to adult worms in mice^39^, our mice infected with 25, 50 and 100 cercariae displayed a burden of around 15, 30 and 60 worms respectively. Based on this and in conjunction with Salem et al. we presume that PET imaging with [^18^F]FDG is not optimal to quantify small numbers of present parasites. This drawback could be solved in the future by using a radiotracer that is directly incorporated by the worms via i.e. oral ingestion. For example, radioactively labeled albumin could be used for the accurate localization of worms and quantification of worm burden, because it was already shown that fluorescence marked albumin is ingested and digested by all development stages of schistosomes^40^.

In principle, however, both studies are consistent in regard to the MRI and PET findings, but only a weak correlation was found between the liver lesions obtained by MRI analysis and the total [^18^F]FDG uptake in livers, portal veins, spleens and abdomen. This underlines the importance of the combined usage of MRI and PET/CT for accurate diagnosis and monitoring of *S. mansoni* infection. We are aware that the high inter-animal variance and the relatively small number of investigated animals per group present a limitation of our study. Therefore, a multimodal imaging study in the same experimental way but with a larger cohort of animals and a higher infection intensity (> 100 cercariae) in a longitudinal approach until beyond 10 weeks after infection would be advantageous or, in order to achieve a more human-like periportal fibrosis low number of cercariae and extended infection time (Andrade ZA, 1997). However, regarding the evaluation of periportal fibrosis or more severe clinical forms, a PET/CT tracer that specifically binds to fibrotic tissue would be required. This would be a great achievement for the future.

In conclusion, this study describes an in vivo scan protocol to characterize *S. mansoni* infection in a mouse model using anatomical MRI imaging and [^18^F]FDG-PET/CT. Morphological MRI imaging is capable to visualize liver tissue alterations during infection of *S. mansoni* and quantification of perioval granulomatous lesions.[^18^F]FDG PET imaging covered changes in the primarily affected organs i.e. livers and spleens as well as the portal veins and abdomen in total and revealed that the uptake of the spleen is a reliable criteria for parasite infection. Due to the observed high inter-animal variance of disease progression it is recommendable to combine PET/CT and MRI imaging to achieve a certain diagnosis and assessment of infection related tissue damage. Moreover, modern imaging techniques like PET/CT and MRI can help to improve the assessment of novel and potentially effective therapeutic approaches.

## Materials and methods

### *Schistosoma mansoni* infection model and study design

*Schistosoma mansoni* (*S. mansoni;* Belo Horizonte strain) was held in a life cycle using *Biomphalaria glabrata* (*B. glabrata*) fresh water snails (Brazilian strain) as intermediate hosts and female NMRI mice as definitive hosts. Six- to eight-weeks-old NMRI mice (Charles River Laboratories, Germany) were housed in an animal facility with a 12:12 h light/dark cycle with ad libitum water and free access to standard chow (SSNIFF, Soest, Netherland). *S. mansoni* cercariae were obtained by mass shedding of 5–10 infected *B. glabrata* after day light exposure. With our experimental design we aim to investigate three widely differing infection intensities and degrees of liver damage^34^. Each mouse was exposed to parasites by sitting in a water bath enriched with 25 (n = 4, light infection), 50 (n = 5, moderate infection) and 100 (n = 5, high infection) *S. mansoni* cercariae for 90 min. At a time when hepatic fibrosis is already established and the different infection intensities are apparent all animals were imaged via MRI (at day 42) and via PET/CT with [^18^F]FDG as radiopharmaceutical at day 43 after infection. Additionally, a healthy control group (n = 3) was imaged at the same days.

### MR imaging

MRI was performed on anesthetized mice (1.5–2.5% isoflurane in oxygen). Animal respiration rate and body temperature were monitored and mice were continuously warmed via a water filled heating pad. The respiration rate was maintained between 35 and 50 breaths/min.

Imaging of the mice was performed using a small animal MRI scanner (BioSpec 70/30, 7.0 T magnetic field strength, 440 mT/m gradient strength, Bruker, Ettlingen, Germany) with a ^1^H transmit resonator (86 mm Resonator, Bruker, Ettlingen, Germany) and a 2-by-2 receive-only surface coil array (Bruker, Ettlingen, Germany) positioned on the abdomen of the mice while the animal laid in supine position. The imaging protocol included morphological T2-weighted TurboRARE sequences to visualize egg granuloma in the liver. Therefore high resolution T2-weighted images of transversal and coronal planes (repetition time: due to respiratory gating approximately 4200 ms; echo time: 26 ms; field of view: coronal: 42 mm × 24 mm; matrix: 351 × 200; voxel size: (0.12 × 0.12 × 0.75) mm^3^; axial: 22.4 mm × 22 mm; matrix: 187 × 183; voxel size: (0.12 × 0.12 × 0.75) mm^3^, 35 to 50 slices depending on mouse size; acquisition time: approx. 10 min per sequence) was performed.

### PET/CT imaging

All PET/CT scans were performed on a small animal PET/CT scanner (Inveon MM-PET/CT, Siemens Medical Solutions, Knoxville, TN, USA) according a standard protocol. Mice were anaesthetized using isoflurane (4% for induction and 1–2.5% maintenance during preparation and scanning) and injected intravenously with a mean dose of 16.72 ± 1.60 MBq [^18^F]FDG via a custom-made micro catheter placed in a tail vein. After an uptake period of 60 min, mice were imaged in prone position for 15 min. During the imaging session, respiration of the mice was controlled and core body temperature was constantly kept at 38 °C via a heating pad. For attenuation correction and anatomical reference whole body CT scans were acquired. Each PET data set was corrected for random coincidences, dead time, scatter and attenuation. CT images were reconstructed with Feldkamp algorithm. PET data were first Fourier rebinned into^41^ a 2D dataset from which real-space images were reconstructed with an ordered subset expectation maximization (OSEM) algorithm with 16 subsets and 4 iterations^41,42^. Data were decay-corrected to the time of injection.

### Image analysis MRI

MR datasets were transferred to PMOD (Version 3.709, PMOD Technologies, Zurich, Switzerland). For image analysis the whole liver was segmented and the volume calculated to detect hepatomegaly due to parasite egg deposition. In a second step, multifocal hyper intense spots around the hepatic lesions, referable to worm egg deposition, were manually counted in three consecutive slices (Fig. 1) and the number of lesions per ten cubic millimeters were calculated.

### Image analysis PET/CT

PET/CT datasets were transferred to the PMOD software. For the analysis of the PET images the CT images were first co-registered by rigid fusion in order to correct the minor motion differences of the animal due to the deep anesthesia breathing or bed translation in the gantry from PET to the CT. Next, on coronal PET images volumes of interest (VOIs) around abdomen, spleen, kidneys, bladder, portal vein and heart were manually drawn with the aid of visual inspection of co-registered CT images (Fig. 4). In the same way, a further VOI (0.2 × 0.2 × 0.2 cm) was placed in the liver of each animal. The VOIs of heart, bladder and kidneys were used to accurately separate the uptakes of [^18^F]-FDG in the VOIs of abdomen (VOI _abdomen_), spleen (VOI _spleen_), portal vein (VOI _portal vein_) and liver (VOI _liver_) from each animal. Quantification of PET data was expressed as mean percent injected dose per cubic centimeter (%ID/cc) for each animal.

### Assessment of infection-related organ alterations

One part of the right lobe of each liver (lobe three) was fixed in 4% neutral buffered formaldehyde solution and embedded in paraffin. Four µm thin sections were stained with hematoxylin/eosin (HE) to differentiate hepatic granulomas from unaffected liver parenchyma. To stain hepatic collagen I/III, liver slices were stained with 0.1% Sirius Red (SR) solution dissolved in aqueous saturated picric acid for 1 h and washed in 0.5% acetic acidified water. Morphometric analysis of granuloma size and of Sirius red positive areas were performed using ImageJ software (v1.47v; National Institute of Health).

### Statistical analyses

Statistical differences in scalar measures from MRI and PET/CT as well biometric data for the different infection groups were determined using One Way ANOVA with the Tukey Test post hoc.. Not normally distributed data or data which failed the equal variance test were tested with Kruskal–Wallis one way analysis of variance on Ranks ([^18^F]FDG uptake of liver and abdomen) and corrected with Dunn’s Method (liver histology data). Correlation of the number of liver lesions and [^18^F]FDG uptakes was assessed using the Pearson product moment correlation coefficient. To quantify the influence of the number of lesions on uptakes regression coefficients were determined. *p* values < 0.05 were considered as significant. All statistical analyses were performed using the SigmaPlot 12.0 software package (Systat Software Inc.).

### Ethical statement

All experimental protocols were performed according to the European Directive 2010/63/EU and German animal protection law (German Tierschutzgesetz), the Tierschutz-Versuchstierverordnung and were approved by the local Animal Research Committee (Landesamt für Landwirtschaft, Lebensmittelsicherheit und Fischerei (LALLF) of the state Mecklenburg-Westernpommerania, file number: LALLF M-V/TSD/7221.3–1-025/16).

## Data Availability

All data generated or analyzed during this study are included in this published article.
